# Effects of Millimolar Steady-State Hydrogen Peroxide Exposure on Inflammatory and Redox Gene Expression in Immune Cells from Humans with Metabolic Syndrome

**DOI:** 10.3390/nu10121920

**Published:** 2018-12-05

**Authors:** Carla Busquets-Cortés, Xavier Capó, Emma Argelich, Miguel D. Ferrer, David Mateos, Cristina Bouzas, Manuela Abbate, Josep A. Tur, Antoni Sureda, Antoni Pons

**Affiliations:** 1 Research Group on Community Nutrition and Oxidative Stress, Science Laboratory of Physical Activity, Department of Fundamental Biology and Health Sciences, University of Balearic Islands, 07122 Palma de Mallorca, Spain; carla_busquets@hotmail.com (C.B.-C.); xaviercapofiol@hotmail.com (X.C.); eargelich15@gmail.com (E.A.); miguel-david.ferrer@uib.es (M.D.F.); david-mateos@hotmail.es (D.M.); cristinabouvel@gmail.com (C.B.); manuela.abbate@uib.es (M.A.); pep.tur@uib.es (J.A.T.); antoni.sureda@uib.es (A.S.); 2 CIBER CB12/03/30038 Fisiopatología de la Obesidad la Nutrición, CIBEROBN, Instituto de Salud Carlos III (ISCIII), University of Balearic Islands, 07122 Palma de Mallorca, Spain

**Keywords:** gene expression, glucose oxidase, hydrogen peroxide, inflammation, mitochondrial biogenesis, neutrophils, PBMCs, ROS

## Abstract

Reactive oxygen species (ROS) such as hydrogen peroxide (H_2_O_2_) can exert opposed effects depending on the dosage: low levels can be involved in signalling and adaptive processes, while higher levels can exert deleterious effects in cells and tissues. Our aim was to emulate a chronic ex vivo oxidative stress situation through a 2 h exposure of immune cells to sustained H_2_O_2_ produced by glucose oxidase (GOX), at high or low production rate, in order to determine dissimilar responses of peripheral blood mononuclear cells (PBMCs) and neutrophils on ROS and cytokine production, and mitochondrial dynamics-related proteins, pro/anti-inflammatory and anti-oxidant gene expression. Immune cells were obtained from subjects with metabolic syndrome. H_2_O_2_ at low concentrations can trigger a transient anti-inflammatory adiponectin secretion and reduced gene expression of toll-like receptors (TLRs) in PBMCs but may act as a stimulator of proinflammatory genes (IL6, IL8) and mitochondrial dynamics-related proteins (Mtf2, NRF2, Tfam). H_2_O_2_ at a high concentration enhances the expression of pro-inflammatory genes (TLR2 and IL1β) and diminishes the expression of mitochondrial dynamics-related proteins (Mtf1, Tfam) and antioxidant enzymes (Cu/Zn SOD) in PBMCs. The GOX treatments produce dissimilar changes in immune cells: Neutrophils were more resistant to H_2_O_2_ effects and exhibited a more constant response in terms of gene expression than PBMCs. We observe emerging roles of H_2_O_2_ in mitochondrial dynamics and redox and inflammation processes in immune cells.

## 1. Introduction

Metabolic syndrome is a constellation of metabolic abnormalities including hypertension, hyperglycaemia, abdominal fatness, and dyslipidemia. Patients suffering from metabolic syndrome exhibit an evident redox imbalance and an inflammatory status that affect the cellular responses and subclasses of immune cells [1,2,3,4]. Peripheral blood leukocyte subclasses from metabolic syndrome patients have a different phenotype compared to non-obese patients, which includes clinical biomarkers that largely reflect already manifested comorbidities [5], and are correlated with subcutaneous adipose tissue macrophages participating in the adipose tissue inflammation [6]. Obesity, a feature of metabolic syndrome, is commonly associated with chronic low-grade inflammation with permanently increased levels of reactive oxygen species (ROS) [7,8,9], such as hydrogen peroxide (H_2_O_2_), superoxide anion (O_2_^·−^), hydroxyl radical (OH^·^), and hypochlorous acid (HClO) [10]. H_2_O_2_, due to its relative stability, mild chemical reactivity, and ability to diffuse [11], is an important metabolite involved in sensing, modulation, and signalling of redox metabolism reactions and processes in the cells [12]. Mounting evidence establishes that these H_2_O_2_–dependent signalling events are triggered by the activation of diverse cell surface receptors [13]. H_2_O_2_ exhibits the ability to specifically oxidise proteins [14,15,16] that activate cascades of downstream responses that lead to various metabolic outputs in the cells [17,18,19,20,21]. According to the downstream pathways that are triggered, homeostatic, pathological, or protective cascades can be activated [22]. Physiological redox signalling is characterized by reversible oxido-reductive modifications [23] of specific cysteine residues, such as thiolate anion (Cys-S-), susceptible to oxidation. The two-electron oxidation of a thiolate by H_2_O_2_ yields sulfenic acid, which is implicated in a number of important biochemical transformations. Cysteines from several transcription factors (e.g., NF-κB), or proteins involved in cell signalling or metabolism (e.g., GAPDH, glutathione reductase, tyrosine phosphatases, kinases, and proteases) can be turned into sulfenic acid [24,25,26]. The reaction of ROS with protein thiols provides a mechanism by which cells can “sense” changes in the redox balance. Understanding the molecular mechanisms and the dosage by which H_2_O_2_ exerts the beneficial/adaptive responses more likely than the negative/pathological ones has far-reaching practical significance since several pathological states including, including metabolic syndrome, are characterized by altered redox biology [16].

To ascertain the role of cellular oxidants in physiological and pathological processes, it is imperative to be able to distinguish and characterize those species involved and monitor its levels in the biological system of interest [27]. Most phenotypic and signalling studies of cellular responses to H_2_O_2_ are designed using extracellular addition, either with a bolus or with sustained generation [28] using glucose oxidase [19]. Peripheral blood mononuclear cells (PBMCs), consisting of T cells, B cells and monocytes, provide an ideal culture model for the study of inflammation and immunity. Neutrophils also serve as cellular models as well considering their various anti-pathogen functions, among others, in an inflammation environment. We aim to emulate a chronic ex vivo oxidative stress situation through the continuous exposure of immune cells, from patients with metabolic syndrome, to H_2_O_2_ produced by glucose oxidase (GOX). The experimental procedure is designed to induce an oxidative stress status in order to distinguish those situations in which H_2_O_2_ generates oxidative imbalance or promotes beneficial responses. We wanted to bring to light the genes whose expression is upregulated when immune cells are exposed to different H_2_O_2_ concentrations. Furthermore, we aimed to evidence the effects of H_2_O_2_ on the expression of the ROS and the cytokine production. 

## 2. Materials and Methods 

### 2.1. Study Design

The present study is a cross-sectional analysis on baseline samples within the frame of the PREDIMED-Plus study, a 6-year multicentre, parallel-group, randomized trial conducted in Spain to assess the effect on cardiovascular disease morbimortality of an intensive weight loss intervention programme based on an energy-restricted traditional MedDiet (erMedDiet), physical activity promotion, and behavioural support, in comparison with a typical care intervention only with energy-unrestricted MedDiet (control group). Details of the study protocol can be found elsewhere [29]. The trial was registered in 2014 at the International Standard Randomized Controlled Trial (ISRCT [30]) with number 89898870.

### 2.2. Participants’ Characteristics

A total of 34 participants from the randomized, multicentre, clinical trial with parallel groups with metabolic syndrome (PREDIMED-Plus) were enrolled in this study. Inclusion criteria included men aged 55–75 and women between 60–75 years old, with a BMI ≥ 27 and < 40 kg/m^2^, that meet at least 3 criteria for metabolic syndrome according to the updated harmonized criteria of the International Diabetes Federation and the American Heart Association and National Heart, Lung, and Blood Institute [31]. Exclusion Criteria were (a) inability or unwillingness to give informed consent, (b) documented history of previous cardiovascular disease, (c) active cancer or a history of malignant tumours in the last 5 years, and (d) impossibility to follow a recommended diet or to carry out physical activity. All participants were informed of the purpose and the implications of the study, and informed consent was obtained from all subjects. All participants were studied at the beginning of the trial; no intervention was yet applied to these subjects. The study was conducted according to the guidelines laid down in the Declaration of Helsinki and all procedures were approved by the Research Ethics Committee of the Balearic Islands (reference no. 2251/14 PI). 

### 2.3. Cell Isolation and Cell Viability Test

Venous blood samples were obtained from the antecubital vein of participants in the study in suitable vacutainers with ethylenediaminetetraacetic acid (EDTA) as an anticoagulant in fasting conditions. Cell counts were determined in an automatic flow cytometer analyzer Technicon H2 (Bayer, Leverkusen, Germany) VCS system. The PBMC and neutrophil fractions were purified from fresh whole blood and isolated following the protocol described previously [32] using Ficoll-Paque PLUS reagent (GE Healthcare, Chalfont St Giles, UK) [33,34]. Briefly, 6 mL of blood was carefully introduced on 4 mL of Ficoll (proportion of 1.5:1) and was then centrifuged at 900× *g*, at 4 °C for 30 min. The plasma and the Ficoll phases were discarded and the PBMCs layer was washed twice with phosphate-buffered saline (PBS), pH 7.4., and centrifuged for 10 min at 1000× *g*, 4 °C. The precipitate containing the erythrocytes and neutrophils was incubated at 4 °C with 0.15 mol/L of ammonium chloride to haemolyse the erythrocytes. The suspension was centrifuged at 750× *g*, at 4 °C for 10 min and the supernatant was then discarded. The neutrophil phase at the bottom was washed first with ammonium chloride and then with PBS. Cell lysates for RNA purification were stored at −80 °C with 1 mL of Tripure Isolation Reagent^®^ (Roche Diagnostics, Mannheim, Germany) until biochemical analysis, while a fresh aliquot was used for ROS determination. Cell viability was assessed using a crystal violet nuclear staining assay [35]. Violet dye binds to proteins and DNA of living cells. Cells that undergo cell death lose their adherence to culture surface and are subsequently lost from the population of cells, reducing the amount of crystal violet staining in a culture. Briefly, 20 µL of 0.5% crystal violet solution in 30% acetic acid was added to 500 µL of suspension of cells and incubate for 10 min at room temperature. Cells were centrifuged (1000× *g*, 10 min) and washed thrice with PBS, until the dye stopped coming off. 100 µL of ethanol were added and all the volume was then transferred onto a 96-well microplate and absorbance at 570 nm was recorded in a microplate reader (Bio-Tek Instruments, Inc., Winooski, VT, USA)

### 2.4. Cell Treatments and Experimental Design

All treatments were performed in 2 mL sterilized tubes containing 3 × 10^6^ PBMCs/mL and 3.5 × 10^6^ neutrophils/mL. Cells were resuspended in 2 mL of RPMI-1640 medium with glucose (2 g/L), l-glutamine and NaHCO_3_. Cells were incubated at 37 °C in a humidified atmosphere for 2 h in the absence and in the presence of two different concentrations (high and low) of glucose oxidase (GOX), following a procedure previously described [36,37]. Lyophilized glucose oxidase (GOX) type X-S from *Aspergillus niger* (~75% protein, 138,370 U/g solid, Sigma-Aldrich) was used to generate H_2_O_2_ (one unit of GOX oxidizes 1.0 μmole of β-D-glucose to D-glucono-δ-lactone and H_2_O_2_ per min at pH 5.1 at 35 °C, equivalent to an O_2_ uptake of 22.4 μL per min, according to the manufacturer’s instructions). GOX was added at concentrations 1 and 0.1 µg solid/mL culture medium (high and low treatment, respectively) in PBMCs. GOX was added at concentrations 15 and 5 µg solid/mL culture medium (high and low treatment, respectively) in neutrophils.

### 2.5. Calibration of H_2_O_2_ to Cell Exposure

**Figure 1:**
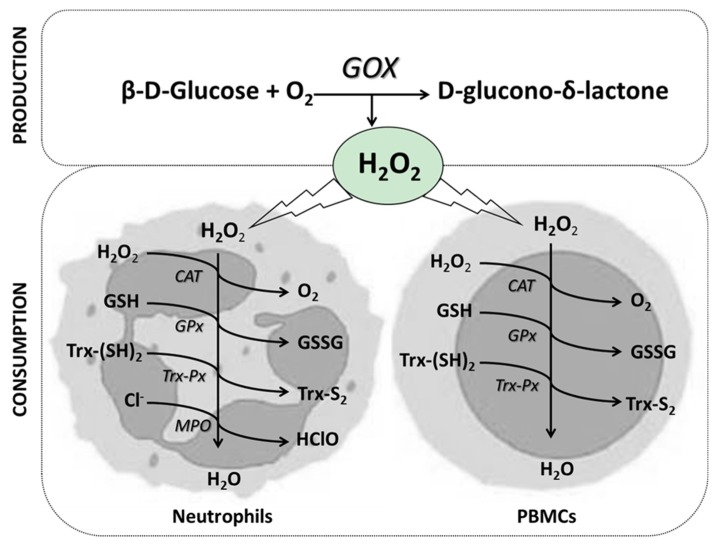
Balance between hydrogen peroxide production and elimination: H_2_O_2_ extracellularly produced by glucose oxidase in the presence of glucose can be intracellularly decomposed by numerous antioxidant systems within the immune cells. GOX: Glucose oxidase; CAT: Catalase; GPx: Glutathione peroxidase; TRx-Px: Thioredoxin peroxidase; MPO: Myeloperoxidase; GSH: Reduced glutathione; GSSG: Oxidized glutathione; Trx(SH)_2_: Oxidized thioredoxin; Trx-S_2_: Reduced thioredoxin; Cl^−^: Chloride anion; HClO: Hypochlorous acid.

The calibration of H_2_O_2_ exposition was monitored colourimetrically in PBMCs and neutrophils cultured in the presence of high or low GOX activities. PBMCs were incubated as indicated above in presence of high (1 µg solid/mL culture medium) or low (0.1 µg solid/mL culture medium) GOX amounts. Neutrophils were incubated as indicated above in presence of high (15 µg solid/mL culture medium) or low (5 µg solid/mL culture medium) GOX amounts. Aliquots of cell culture were taken at different incubation times, and deproteinized with acetone [38,39] at 4 °C in order to stop the H_2_O_2_ production by the enzyme. The H_2_O_2_ levels were determined in the deproteinization supernatants (centrifugation conditions: 900× *g* at 4 °C for 5 min) by using horseradish peroxidase and tetramethylbenzidine (TMB) and measuring the absorbance at 450 nm [36,40]. H_2_O_2_ concentration was calculated with a standard curve of known concentrations. Figure 1 summarizes the process to attain the steady state of H_2_O_2_ used in our experimental model. The steady state is a consequence of the extracellular production of hydrogen peroxide by glucose oxidase in the presence of glucose, and of the elimination of H_2_O_2_ through the enzymatic mechanisms within the neutrophils and PBMCs. When the steady state is attained, the rate of production and elimination is equal.

### 2.6. Stimulated PBMCs and Neutrophils H_2_O_2_ Production

After the 2 h incubation at 37 °C, H_2_O_2_ production by PBMCs and neutrophils was measured using 2,7-dichlorofluorescin-diacetate (DCFH-DA) as an indicator [41,42]. A stock solution of DCFH-DA (2.05 mM) in ethanol was prepared and stored at −20 °C until analysis. Hanks’ Balanced Salts Medium was prepared in a relation of 9.8 g/L water. H_2_O_2_ production in PBMCs and neutrophils was measured before and after stimulation with lipopolysaccharide (LPS) (100 µg/mL PBS) from *Escherichia coli* (Sigma-Aldrich, St. Louis, MO, USA) or Zymosan A (ZYM) (1 mg/mL PBS) from *Saccharomyces cerevisiae* (Sigma-Aldrich). A total of 50 µL of LPS or ZYM prepared in PBS was added to the wells. DCFH-DA in ethanol was diluted in Hanks’ Balanced Salts Medium (relation 30 µL DCFH-DA/mL Hanks’) and added to a 96-well microplate containing 50 µL of cell suspension (containing about 6 × 10^5^ cells). The fluorescence (Ex, 480 nm; Em, 530 nm) was recorded at 37 °C for 60 min in FLx800 Microplate Fluorescence Reader (Bio-tek Instruments, Inc.) by punctual ultraviolet light exposures and emission readings were recorded every minute (60 total readings). H_2_O_2_ concentration was calculated by measuring fluorescence of a standard curve of known H_2_O_2_ concentration after its reaction with DCFH-DA, in the same conditions as the samples.

### 2.7. RNA Isolation and mRNA Gene Expression

mRNA expressions were determined by Real Time PCR (RT-PCR) based on the incorporation of a fluorescent reporter dye and using human 18S ribosomal as the reference gene [43]. For this purpose, the total mRNA from PBMCs and neutrophils was isolated by extraction with Tripure Isolation Reagent^®^ (Roche Diagnostics, Mannheim, Germany) following a procedure previously described [44,45]. cDNA was synthesized from 1 µg total RNA using 50 units of Expand Reverse Transcriptase (Roche Diagnostics, Germany) and 20 pmol of oligo (dT) for 60 min at 37 °C in a final volume of 10 μL, according to the manufacturer’s instructions. Semiquantitative PCR of the resulting cDNA (3 μL) was amplified using the LightCycler^®^ 480 instrument (Roche Diagnostics, Mannheim, Germany) with FastStart DNA MasterPLUS SYBR™ Green I kit (Roche Diagnostics, Mannheim, Germany). The specific primers and amplification conditions used for each gene are presented in Table 1. Target cDNAs were amplified as follows: one cycle at 95 °C for 10 min, followed by 45 cycles of amplification (95 °C for 10 s; specific annealing temperature for 10 s; 72 °C for 15 s). mRNA levels in the control situation (no glucose oxidase in the medium) were arbitrarily referred to as 1.

### 2.8. Cytokine Determination

Adiponectin, IL6, and TNFα from cell incubation supernatants were determined using individual Human High Sensitivity ELISA kits (Diaclone, Besaçon, Cedex, France) following the manufacturer’s instructions. Intra-assay and inter-assay reproducibility were < 5% and < 10%, respectively. The rate of cytokine production was calculated by its determination in the cell culture after 2 h of incubation. The amount of cytokine present divided by the counts of PBMCs and by the time of incubation was considered the rate of cytokine production (pg/min/10^6^).

### 2.9. Statistical Analysis

Statistical analysis was carried out using Statistical Package for Social Sciences (SPSS v.21.0 for Windows0 (IBM Software Group, Chicago, IL, USA). Results in figures and tables are expressed as mean ± SEM and *p* values < 0.05 were considered statistically significant. Student’s t-test for paired data was used to determine the effects of the GOX treatments (high GOX treatments with respect to control, and low GOX treatments with respect to control) in gene expression and cytokine production. One-way ANOVA was performed to determine differences in H_2_O_2_ production between groups and cell viability between treatments. 

## 3. Results

Participants in the study were 34 men and women with documented metabolic syndrome. Anthropometric and haematological characteristics are shown in Table 2. The participants were obese (BMI higher than 30) with values of glucose, cholesterol, and triglycerides borderline to pathological cut off values. The blood cell counts were into the normal distribution of healthy subjects.

**Figure 3:**
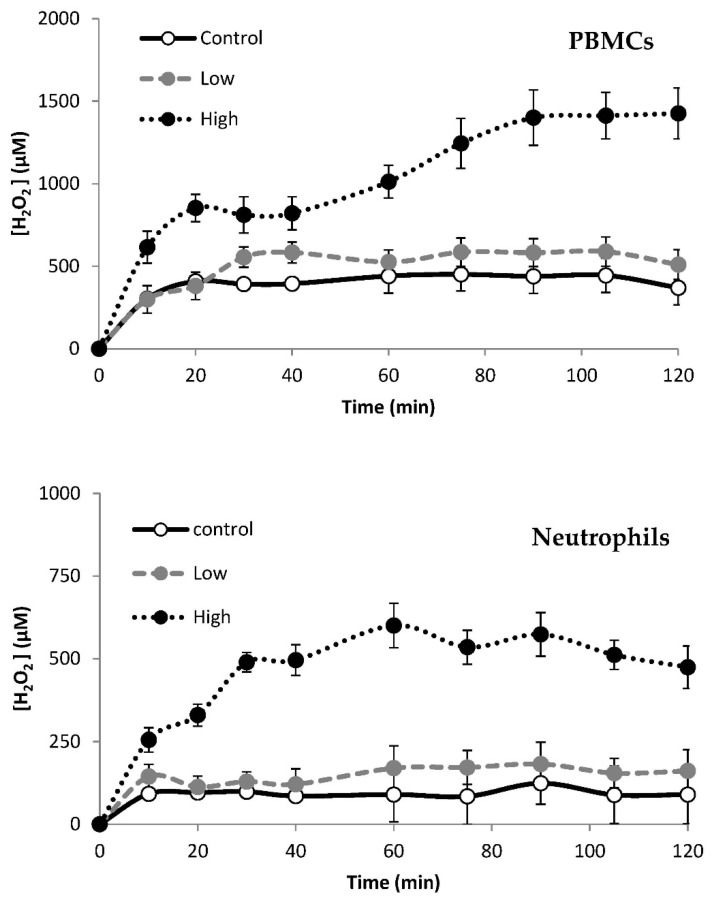
Levels of H_2_O_2_ in the culture medium of PBMCs and neutrophils in the control and after high and low H_2_O_2_ exposure by GOX addition for 2 h at 37 °C. PBMCs: High indicates H_2_O_2_ production rate by 1 µg solid GOX/mL culture medium and Low indicates H_2_O_2_ production rate by 0.1 µg solid GOX/mL culture medium. Neutrophils: High indicates H_2_O_2_ production rate by 15 µg solid GOX/mL culture medium and Low indicates H_2_O_2_ production rate by 5 µg solid GOX/mL culture medium. Control indicates PBMCs or neutrophils are cultured in the medium without GOX addition.

Figure 3 shows the progression at different times of H_2_O_2_ levels in the immune cells culture medium in the control and after the addition of high and low GOX. The cell counts in the cellular culture were the same as in blood (3.1 ± 0.2 × 10^3^ PBMCs/mm^3,^ and 3.9 ± 0.3 × 10^3^ neutrophils/mm^3^). When no GOX was added to the culture medium (control situation), PBMCs attained a H_2_O_2_ steady state at 400 ± 60 µM, while neutrophils attained steady-state H_2_O_2_ levels at 95 ± 8 µM. The H_2_O_2_ steady state was reached within the first 10 min of PBMCs or neutrophils incubation and it is stable for 2 h. The concentration of H_2_O_2_ in the culture medium reaches the steady state levels at different times and levels depending on the dosage of GOX applied and on the cell type. Neutrophils reach the steady state after 30 min of high GOX addition, while for PBMCs it takes almost 70 min. The steady state was attained after 10 min by neutrophils and after 30 min by PBMCs in the case of low GOX exposure. The H_2_O_2_ steady state levels were different for neutrophils and for PBMCs at low and high GOX. The stationary state H_2_O_2_ levels were 1400 ± 154 µM with high GOX concentration (1 µg solid GOX/mL culture media) in PBMCs whereas the steady state levels for neutrophils with high GOX concentration (15 µg solid GOX/mL culture medium) were 500 ± 50 µM. The stationary state H_2_O_2_ levels were 500 ± 75 µM with low GOX concentration (0.1 µg solid GOX/mL culture medium) in PBMCs whereas the steady state levels for neutrophils with low GOX concentration (5 µg solid GOX/mL culture medium) was 180 ± 40 µM. 

The continuous H_2_O_2_ production at a high or low rate by GOX addition to the culture medium significantly influenced the PBMCs and neutrophils H_2_O_2_ production following LPS or ZYM stimulation (Table 3). Neutrophils produced H_2_O_2_ on a cellular basis at a higher rate than PBMCs, under both LPS and ZYM stimuli and even previously or after the treatment with high or low H_2_O_2_ (significances not shown). The H_2_O_2_ production rate was higher under ZYM stimuli than LPS stimuli, in both PBMCs and neutrophils and independently of the H_2_O_2_ concentration (significances not shown). Previous incubation of PBMCs with high or low GOX dosages significantly decreased the rate of H_2_O_2_ production after ZYM but not after LPS stimulation. No significant effects were found when neutrophils previously incubated in the presence of GOX were stimulated with ZYM. However, when LPS was used as stimulus, high GOX concentration exposure significantly augmented H_2_O_2_ production with respect to the control. 

The effects of 2 h of incubation with GOX on the production of cytokines by PBMCs and neutrophils were analyzed (Table 4). The rate of adiponectin production in PBMCs [46] significantly increased only after high GOX dosage. On the contrary, both GOX treatments provoked a significant decrease in IL-6 production, while TNFα levels remained constant, independent of the concentration of GOX applied studied in PBMCs. 

In neutrophils, no adiponectin production was detected at all, confirming the inability of this cellular type to synthesize this adipokine. IL-6 and TNFα production remained changeless under H_2_O_2_ exposure in neutrophils. 

**Figure 4:**
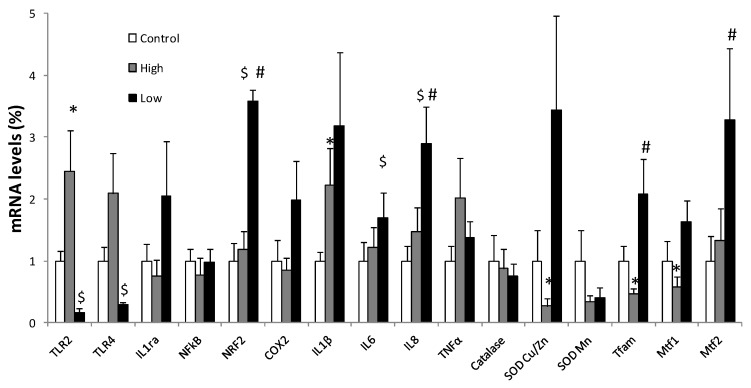
Relative gene expression of pro/anti-inflammatory proteins, antioxidant enzymes and mitochondrial dynamics related proteins in PBMCs. Ribosomal 18S as a reference. Control was arbitrarily referred to as 1. (Control: only cells present in the medium; high: 1 µg solid GOX/mL; low: 0.1 µg solid GOX/mL). Statistical analysis: Student’s t-test for paired data, *p* < 0.05. (*) Significant effects between the high treatment and the control; ($) significant effects between the low treatment and the control; (#) Significant effects between the high treatment and the low treatment.

Changes in gene expression in PBMCs and neutrophils were studied after continuous exposure to two different concentrations of H_2_O_2_ produced by two different dosages of GOX (Figure 3 and Figure 4). The expression of genes encoding immune and inflammatory-related proteins (COX2, NFkB, TNFα, IL6, IL1β, IL1α, IL8, TLR2, TLR4), antioxidant enzymes (CAT, SOD Cu/Zn, SOD Mn), and mitochondrial dynamics-related proteins (Mtf1, Mtf2, Tfam, and NRF2) were analysed. 

In PBMCs (Figure 4) high H_2_O_2_ exposure significantly increased the expression of immune and inflammatory-related proteins as IL1β and TLR2, while it provoked a decrease in the expression of SOD Cu/Zn, Mtf1 and Tfam. Low H_2_O_2_ exposure promoted a rise in the expression of mitochondrial proteins Tfam, NRF2, and Mtf2 and proinflammatory mediators IL6 and IL8, while it diminished the expression of receptors TLR2 and TLR4. Significant differences between the two high and low GOX treatments were also detected in IL8, NRF2, and Tfam that increased their mRNA expression in low with respect to high GOX treatments.

**Figure 5:**
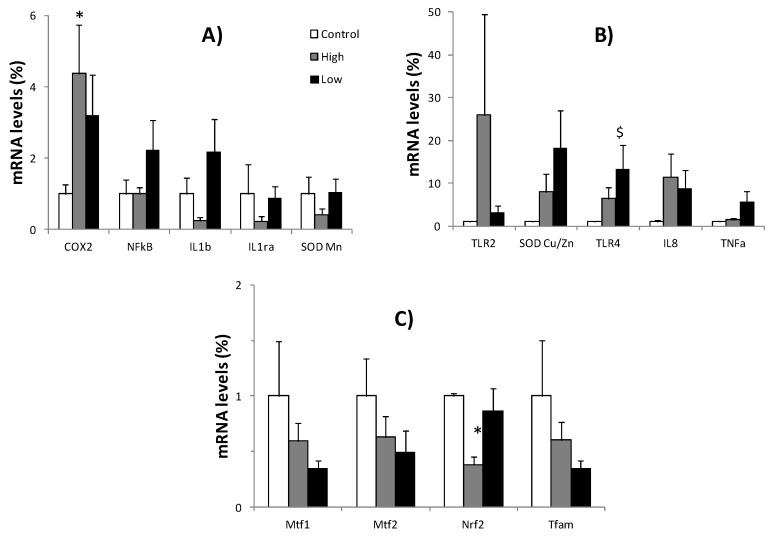
Relative expression of pro/anti-inflammatory proteins, antioxidant enzymes (**A**,**B**), and mitochondrial dynamics related proteins (**C**) in neutrophils Ribosomal 18S as a reference (Control: Only cells present in the medium; High: 15 µg solid GOX/mL; Low: 5 µg solid GOX/mL). Statistical analysis: Student’s t-test for paired data, *p* < 0.05. (*)Significant effects between the high treatment and the control; ($) significant effects between the low treatment and the control.

In neutrophils (Figure 5), the continuous production of high or low extracellular H_2_O_2_ exerted a lower influence than in PBMCs on the expression of inflammatory, mitochondrial dynamics, and antioxidant genes. In fact, a low and high rate of H_2_O_2_ production maintained the control expression of inflammatory genes, such as NFkB, TNFα, IL1rα, IL1β, IL6, IL8, and TLR2, and the control expression of antioxidant and mitochondrial dynamics genes such as SOD Cu/Zn, SODMn, Mtf1, Mtf2, and Tfam. Only the expression of COX2, TLR4, and NRF2 evidenced a significant influence of the exposition to high or low H_2_O_2_ production in neutrophils. High H_2_O_2_ production significantly increased the COX2 expression but decreased the expression of NRF2, whereas the low H_2_O_2_ production significantly increased the expression of TLR4 in neutrophils. 

## 4. Discussion

A relevant feature of this study is that neutrophils, in spite of being stimulated with higher GOX concentrations than PBMCs, apparently exhibit a steady state of H_2_O_2_ levels three times lower than PBMCs during exposure to both GOX concentrations. Furthermore, neutrophils reach the steady state three times faster than PBMCs. These facts reveal a higher capacity of neutrophils to eliminate H_2_O_2_ than PBMCs [47]. The method developed is useful to maintain cells to an extracellular sustained production of H_2_O_2_ and to reach a steady state of H_2_O_2_ levels in the culture media in less than 2 h. Phagocytes (neutrophils, macrophages, monocytes, and eosinophils) contribute to the plasma H_2_O_2_ pool [48], whereas the H_2_O_2_ generated in the plasma or on the surface of cells can actually enter cells, through aquaporins [49], where there are several enzymes that can eliminate it very rapidly [50]. We observe that isolated PBMCs or neutrophils in culture produce and eliminate H_2_O_2_ to quickly attain a cell-dependent steady state. It is also relevant that both PBMCs and neutrophils in culture reach a steady state at a micromolar H_2_O_2_ level, being higher in PBMCs than in neutrophils. We also observe that the capabilities of H_2_O_2_ production by PBMCs under stimulation with ZYM or LPS are in a similar order to Neutrophils when no GOX is added. Thus, the capabilities to eliminate H_2_O_2,_ taking into account the H_2_O_2_ scavenging enzyme activities, are higher in neutrophils than in PBMCs [51,52,53]. These characteristics are coincident with a H_2_O_2_ steady state at higher H_2_O_2_ levels in PBMCs than neutrophils. The H_2_O_2_ steady state in human blood is difficult to determine but it ranges from a possible low of 0.25 μM to a probable normal range of 1–5 μM, and a high range of 30–50 μM in certain disease states or during chronic inflammation [48]. The H_2_O_2_ steady state in PBMCs or neutrophils in culture in our study are about 400 μM or 95 μM respectively, being higher than in plasma, probably due to the lack of plasma scavenging antioxidant enzymes in the culture media such as plasma catalase. Basal cytosolic steady-state H_2_O_2_ concentrations are estimated to be lying in the low nanomolar range (≈ 1–10 nM) [20], while during oxidative signalling events H_2_O_2_ levels can rise transiently to the upper nanomolar range (≈ 500–700 nM) [54]. The intracellular H_2_O_2_ levels in PBMCs and neutrophils are not determined in the present experience but the H_2_O_2_ steady state attained in the culture using different GOX levels is at micromolar level. Despite the fact that exogenous in vivo H_2_O_2_ concentrations rarely exceed the low-micromolar range (up to 10 µM), studies can be performed with H_2_O_2_ levels in the upper micromolar or even millimolar range to obtain cellular responses [28]. The capabilities to scavenge H_2_O_2_ by PBMCs and neutrophils are very high and this fact could prevent the intracellular H_2_O_2_ from rising to toxicological levels, at least in the control and low H_2_O_2_ steady state levels. In fact, in all cases, we do not detect any effects of H_2_O_2_ on the cell viability. This is in accordance with a low rate of oxidative protein damage in neutrophils after oxidative insults as hypoxia/reoxygenation in apnoea diving [55] or after acute intense physical activity in trained athletes [56].

An optimal level of inflammation is required for immunity enhancement while chronic inflammation is associated with several metabolic disorders like type 2 diabetes, overweight, and obesity. Indeed, the latter is characterized by chronic low-grade inflammation with permanently increased oxidative stress [7,8,57,58]. This pathological condition normally leads to low levels of plasmatic adiponectin (with anti-inflammatory properties) and high levels of TNFα and IL6 (pro-inflammatory) [59]. Adiponectin is one of the most abundantly secreted adipose tissue proteins that is negatively correlated with obesity; the expression of the anti-inflammatory adiponectin is reduced in adipose tissue of obese individuals compared to lean individuals [60,61]. Previous research showed that adiponectin can reduce the secretion of markers involved in the activation of NFκB including TNF-α, IL-6 by adipocytes and macrophages through TLRs [62]. We observed that IL6 production decreases while the adiponectin secretion concurrently increases as a result of exposure to H_2_O_2_ in PBMCs. This fact could be related to direct or indirect anti-inflammatory proprieties of H_2_O_2_ [63,64,65]. We also evidence that PBMCs secrete adiponectin, as documented before, [46] and it is increased by the different GOX treatments.

The observed increase in H_2_O_2_ production by immune cells after immunostimulatory exposure could be a multifactorial response derived from the increased cellular metabolism induced by the immune activation, and it is probably cell type-dependent. The higher rate of production of H_2_O_2_ observed in neutrophils than in PBMCs after LPS or ZYM stimulation, could be a consequence of the two mitochondrial and lysosomal pathways of oxidant species generation in neutrophils [66,67,68] versus the only mitochondrial pathway in PBMCs [69,70]. LPS exerts its action mainly through interaction with TLR4 [71] whereas ZYM directly binds TLR2 [72]. The H_2_O_2_ production is higher under ZYM stimuli than LPS stimuli, in both PBMCs and neutrophils, likely indicating a higher reception by TLR2 than the TLR4 way. However, the effects of the GOX treatments on the expression of TLR2 and TLR4 in PBMC and neutrophils are not coincident with the effects of GOX treatments on H_2_O_2_ production after immune stimulation. It may be possible to internalize the receptor TLR4 in the membrane in a physiological situation with high H_2_O_2_ production rate, such as acute physical activity [73], and, thus, make the TLRs less available for immunostimulators recognition. The observed effects after the LPS and ZYM immune stimulation in terms of ROS production could also be attributable to an action subsequent to the interaction of immunostimulators with TLRs receptors such as the activation of the NFκβ pathway or even at mitochondrial or lysosomal levels [74].

The results obtained reveal that the 2 h exposure of PBMCs and neutrophils to different levels H_2_O_2_ produced by a high and low dosage of GOX from patients with metabolic syndrome induce dissimilar changes in these cellular types, with a more resistance of neutrophils to H_2_O_2_ effects in terms of gene expression than PBMCs. In PBMCs, expression of proinflammatory proteins IL6, IL8, and IL1β augment after the 2 h incubation with GOX; this pattern in PBMCs suggests that chronic exposure to H_2_O_2_, either high or low production rate, promotes a transient inflammatory response in immune cells. Mtf2, a mediator of mitochondrial fusion, also exhibits increased expression after H_2_O_2_ incubation, pointing out an emerging role of mitochondrial dynamics-related proteins in inflammation processes. Indeed, it has been reported that Mtf2 interacts with NLRP3 and enhances inflammasome activation, a multiproteic complex responsible for activation for inflammatory processes [75]. Mounting evidence suggests that the involvement of Mtf2 in multiple signalling pathways is not only restricted to the regulation of mitochondrial shape [76]. 

H_2_O_2_ exposure at low GOX dosage activates the expression of Mtf2 and Tfam in PBMCs and puts in evidence the induction of mitochondrial remodelling and biogenesis by H_2_O_2_. Conversely, high H_2_O_2_ exposure reduces the gene expression of Mtf1 and Tfam in PBMCs. The mitochondrial biogenesis might be considered as an antioxidant mechanism to avoid oxidative stress and also a protective quality-control process for mitochondria [77,78,79]; mitochondria being one of the main sites of ROS production. Mitochondrial fusion and fission in conjunction with mitochondrial autophagy preserve and control organelle quality [80]. 

The antioxidant enzymatic mechanisms are apparently unaffected in neutrophils and lightly inhibited after H_2_O_2_ exposure in PBMCs. The effects of H_2_O_2_ on the antioxidant enzymes expression are dependent on the dosage of H_2_O_2_ [36]. PBMCs exhibit a decrease in the expression of Cu/Zn SOD at high H_2_O_2_ exposure while the other antioxidant enzymes are not affected. Neutrophils seem to be more resistant to the effects of H_2_O_2_ on the gene expression of antioxidant enzyme and mitochondrial proteins than PBMCs. Neutrophils are more resistant cells towards oxidative damage than PBMCs [81,82,83]; in fact, we had to apply higher glucose oxidase concentrations to these cell types to induce oxidative stress. The treatment with high GOX reduces the expression of NRF2 in neutrophils. NRF2, also called GABPA [84], functions as a transcription factor that activates the expression of some key metabolic genes regulating cellular growth and nuclear genes required for mitochondrial respiration as well as mitochondrial biogenesis, DNA transcription and replication [85,86,87]. The decreased NRF2 expression induced by high H_2_O_2_ exposure could influence mitochondrial respiration in neutrophils. In addition, the treatment with high H_2_O_2_ increases the expression of COX2, an inducible enzyme responsible for synthesizing pro-inflammatory prostaglandins from araquidonic acid and resolving-inflammation molecules as resolvins from eicosapentaenoic and docosahexaenoic acid [88,89].

This fact put in evidence a possible pro-inflammatory effect of exposure to high levels of H_2_O_2_ in neutrophils.

To sum up, the dosage of exposure to H_2_O_2_ seems to represent a key variable that influences the side of the double-edged role of this oxidant species at any given moment: Different H_2_O_2_ extracellular levels influence the pro/anti-inflammatory, pro/antioxidant, and mitochondrial status in the cells. Therefore, a close control of these levels may have medical relevance in terms of diagnosis/prognosis of diseases with altered inflammatory and oxidative status as metabolic syndrome. 

## 5. Conclusions

Neutrophils exhibit higher capacity than PBMCs to eliminate H_2_O_2_ extracellularly produced by glucose oxidase in a medium with glucose. The exogenous H_2_O_2_ exposure of immune cells from patients with metabolic syndrome induce dissimilar changes in these cellular types, with a greater resistance of neutrophils to H_2_O_2_ effects in terms of gene expression than PBMCs. Indeed, H_2_O_2_, constantly produced (e.g., for 2 h) and ex vivo controlled, triggers a transient anti-inflammatory adipokine secretion in PBMCs but acts as a genetic stimulator of proinflammatory genes in both PBMCs and neutrophils. Antioxidant defences are downregulated by high H_2_O_2_ levels in PBMCs but cushioned in neutrophils. H_2_O_2_ influences on mitochondrial dynamics related protein gene expression. At a low production rate, H_2_O_2_ promotes biogenesis and remodelling mitochondria which might be considered as a hormetic protective quality-control process towards oxidative stress; meanwhile, a high H_2_O_2_ production rate induces the downregulation of mitochondrial biogenesis and structural remodelling. A close control of H_2_O_2_ levels may have medical relevance in pathological processes with altered inflammatory and oxidative status as metabolic syndrome.

## Figures and Tables

**Figure 2:**
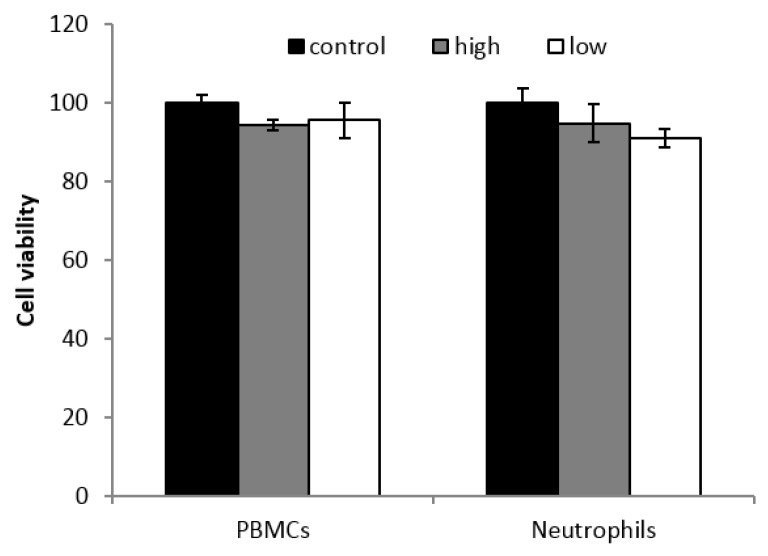
Peripheral blood mononuclear cells (PBMCs) and neutrophils viability after 2 h of exposure to a high and low dosage of H_2_O_2_ at 37 °C. Statistical analysis: One way ANOVA, *p* < 0.05. PBMCs: 1 and 0.1 µg solid GOX/mL (high and low, respectively). Neutrophils: 15 and 5 µg solid GOX/mL (high and low, respectively). Control: no GOX added.

**Table 1:** Primer sequence and annealing temperatures used for the real-time PCR.

Gene	Primer		Temp
**18S**	Fw:	5′-GACTCAACACGGGAAACCCTCAC-3′	60 °C
	Rv:	5′-GACTCAACACGGGAAACCCTCAC-3′	
**COX2**	Fw:	5′-TTGCCTGGCAGGGTTGCTGGTGGTA-3′	67 °C
	Rv:	5′-CATCTGCCTGCTCTGGTCAATGGAA-3′	
**CAT**	Fw:	5′-TTT GGC TAC TTT GAG GTC AC-3′	60 °C
	Rv:	5′-TCC CCA TTT GCA TTA ACC AG-3′	
**TNFα**	Fw:	5′-CCCAGGCAGTCAGATCATCTTCTCGGAA-3′	63 °C
	Rv:	5′-CTGGTTATCTCTCAGCTCCACGCCATT-3′	
**IL6**	Fw:	5′-TACATCCTCGACGGCATCTC-3′	63 °C
	Rv:	5′-ACTCATCTGCACAGCTCTGG-3′	
**IL1β**	Fw:	5′-GGACAGGATATGGAGCAACA-3′	58 °C
	Rv:	5′-GGCAGACTCAAATTCCAGCT-3′	
**IL8**	Fw:	5′-GCTCTGTGTGAAGGTGCAGTTTTGCCAA-3′	63 °C
	Rv:	5′-TGAACATGGGGAGTGTTTCA-3′	
**NFkB**	Fw:	5′-AAACACTGTGAGGATGGGATCTG-3′	60 °C
	Rv:	5′-CGAAGCCGACCACCATGT-3′	
**IL10**	Fw:	5′-AGAACCTGAAGACCCTCAGGC-3′	58 °C
	Rv:	5′-CCACGGCCTTGCTCTTGTT-3′	
**Mfn1**	Fw:	5′-TGTTTTGGTCGCAAACTCTG-3′	60 °C
	Rv:	5′-CTGTCTGCGTACGTCTTCCA-3′	
**Mfn2**	Fw:	5′-ATGCATCCCCACTTAAGCAC-3′	60 °C
	Rv:	5′-CCAGAGGGCAGAACTTTGTC-3′	
**Tfam**	Fw:	5′-CAAGACAGATGAAACCACCTC-3′	60 °C
	Rv:	5′-AGATTGGGGTCGGGTCACT-3′	
**NRF2**	Fw:	5′-GCGACGGAAAGAGTATGAGC-3′	60 °C
	Rv:	5′-GTTGGCAGATCCACTGGTTT-3′	
**TLR2**	Fw:	5′-GGGTTGGAAGCACTGGACAAT-3′	55 °C
	Rv:	5′-TTCTTCCTTGGAGAGGCTGA-3′	
**TLR4**	Fw:	5′-GGTCACCTTTTCTTGATTCCA-3′	55 °C
	Rv:	5′-TCAGAGGTCCATCAAACATCAC-3′	
**SOD Cu/Zn**	Fw:	5′-TCA GGA GAC CAT TGC ATC ATT-3′	63 °C
	Rv:	5′-CGC TTT CCT GTC TTT GTA CTT TCT TC-3′	
**SOD Mn**	Fw:	5′-GAGAAGGTACCAGGAGGCGTTG-3′	64 °C
	Rv:	CAAGCCAACCCCAACCTGAGC-3′	

18S: Ribosomal 18S; COX2: Cyclooxigenase 2; CAT: catalase; TNFα: Tumor necrosis factor alpha; IL6: Interleukin 6; IL1β: Interleukin 1β; IL8: Interleukin 8; NFkB: Nuclear factor kappa-light-chain-enhancer of activated B cells; IL10: Interleukin 10; Mfn1, Mitofusin 1; Mfn2, Mitofusin 2; Tfam: Transcription factor A, mitochondrial; NRF2: Nuclear respiratory factor 2; TLR2: toll-like Receptor 2; TLR4: Toll-like receptor 4; SOD Cu/Zn: Copper/zinc superoxide dismutase; SOD Mn: manganese superoxide dismutase.

**Table 2:** Anthropometric and haematological characteristics of the participants.

	(*N* = 34)	Reference Value
Age (years)	64.2 ± 0.7	^1^
Weight (kg)	85.5 ± 1.9	^1^
Height (height)	164.3 ± 1.4	^1^
Waist circumference (cm)	107.5 ± 1.3	^1^
BMI (kg/m^2^)	31.6 ± 0.5	^1^
Glucose (mg/dL)	112.6 ± 3.1	76–110
Triglycerydes (mg/dL)	155.9 ± 9.1	10–150
Total cholesterol (mg/dL)	198.8 ± 6.1	<200
PBMCs (10^3^ cells/mm^3^)	3.1 ± 0.2	^1^
Lymphocytes (10^3^ cells/mm^3^)	2.5 ± 0.1	1–5
Monocytes (10^3^ cells/mm^3^)	0.6 ± 0.01	0–0.8
Neutrophils (10^3^ cells/mm^3^)	3.9 ± 0.3	1.8–7.7

Cell viability after GOX addition, and consequent H_2_O_2_ generation in the culture medium, was assessed. Results are shown in Figure 2. No significant changes were observed in cell viability when cells were treated with high and low GOX concentrations with respect to control conditions (no GOX present in the medium). ^1^ No reference value for this parameter.

**Table 3:** H_2_O_2_ production by PBMCs and neutrophils stimulated with LPS or ZYM after high and low H_2_O_2_ treatments.

			Control	High	Low
nmol H_2_O_2_/min/10^6^ cells	PBMCs	ZYM	21.8 ± 3.2	14.6 ± 2.3 *	17.0 ± 2.8 $
		LPS	63.3 ± 2.7	9.0 ± 2.2	8.7 ± 3.1
	Neutrophils	ZYM	62.9 ± 9.3	62.9 ± 8.3	63.3 ± 7.4
		LPS	17.4 ± 2.4	49.1 ± 7.4 *	27.5 ± 5.5

High and Low indicates high and low rate of H_2_O_2_ production by high and low level of Glucose oxidase in the medium. Statistical analysis: One way ANOVA, *p* < 0.05. (*) significant effects between the high treatment and the control; ($) significant effects between the low treatment and the control; LPS: lipopolysaccharide; ZYM: zymosan.

**Table 4:** Cytokine levels in supernatants of PBMCs and neutrophils samples after 2 h continuous exposure to high and low GOX treatments.

	(pg/min/10^6^)	Control	High	Low
PBMCs	Adiponectin	61.1 ± 11.9	92.1 ± 14.7 *	83.6 ± 15.7
	IL-6	116 ± 51	12.7 ± 4.7 *	38.8 ± 24.6 $
	TNFα	1587 ± 220	1643 ± 264	1506 ± 172
Neutrophils	Adiponectin	ND	ND	ND
	IL-6	38.3 ± 11.41	31.1 ± 8.6	46.8 ± 15.7
	TNFα	374 ± 90	382 ± 112	406 ± 64.6

High and Low indicates high and low rate of H_2_O_2_ production by high and low level of Glucose oxidase in the medium. Statistical analysis: Student’s t-test for paired data, *p* < 0.05. (*) Significant effects between the high treatment and the control; ($) significant effects between the low treatment and the control. ND: Non-detected.
